# Use of autologous ^99m^Technetium-labelled neutrophils to quantify lung neutrophil clearance in COPD

**DOI:** 10.1136/thoraxjnl-2018-212509

**Published:** 2019-01-23

**Authors:** Nicola Tregay, Malcolm Begg, Anthony Cahn, Neda Farahi, Kathryn Povey, Sujith Madhavan, Rosalind Simmonds, Daniel Gillett, Chandra Solanki, Anna Wong, Joanna Maison, Mark Lennon, Glyn Bradley, Emily Jarvis, Marius de Groot, Fred Wilson, Judith Babar, A Michael Peters, Edith M Hessel, Edwin R Chilvers

**Affiliations:** 1 Department of Medicine, University of Cambridge, Cambridge, UK; 2 Refractory Respiratory Inflammation DPU, Respiratory TAU, GlaxoSmithKline, Stevenage, UK; 3 Discovery Medicine, Respiratory TAU, GlaxoSmithKline, Stevenage, UK; 4 Clinical Pharmacology Science and Study Operations, GlaxoSmithKline, Stockley Park, UK; 5 Clinical Unit Cambridge, GlaxoSmithKline, Cambridge, UK; 6 Department of Nuclear Medicine, Cambridge University Hospitals, Cambridge, UK; 7 Target Sciences, GlaxoSmithKline, Stevenage, UK; 8 Biostatistics, GlaxoSmithKline, Stevenage, UK; 9 Experimental Medicine Unit, Immunoinflammation TAU, GlaxoSmithKline, Stevenage, UK; 10 Department of Radiology, Cambridge University Hospitals, Cambridge, UK; 11 Division of Clinical and Laboratory Investigation, Brighton and Sussex Medical School, Brighton, UK

**Keywords:** imaging/ct mri etc, innate immunity, neutrophil biology, copd pathology

## Abstract

**Rationale:**

There is a need to develop imaging protocols which assess neutrophilic inflammation in the lung.

**Aim:**

To quantify whole lung neutrophil accumulation in (1) healthy volunteers (HV) following inhaled lipopolysaccharide (LPS) or saline and (2) patients with COPD using radiolabelled autologous neutrophils and single-photon emission computed tomography/CT (SPECT/CT).

**Methods:**

20 patients with COPD (Global initiative for chronic obstructive lung disease (GOLD) stages 2–3) and 18 HVs were studied. HVs received inhaled saline (n=6) or LPS (50 µg, n=12) prior to the injection of radiolabelled cells. Neutrophils were isolated using dextran sedimentation and Percoll plasma gradients and labelled with ^99m^Technetium (Tc)-hexamethylpropyleneamine oxime. SPECT was performed over the thorax/upper abdomen at 45 min, 2 hours, 4 hours and 6 hours. Circulating biomarkers were measured prechallenge and post challenge. Blood neutrophil clearance in the lung was determined using Patlak-Rutland graphical analysis.

**Results:**

There was increased accumulation of ^99m^Tc-neutrophils in the lungs of patients with COPD and LPS-challenged subjects compared with saline-challenged subjects (saline: 0.0006±0.0003 mL/min/mL lung blood distribution volume [mean ±1 SD]; COPD: 0.0022±0.0010 mL/min/mL [p<0.001]; LPS: 0.0025±0.0008 mL/min/mL [p<0.001]). The accumulation of labelled neutrophils in 10 patients with COPD who underwent repeat radiolabelling/imaging 7–10 days later was highly reproducible (0.0022±0.0010 mL/min/mL vs 0.0023±0.0009 mL/min/mL). Baseline interleukin (IL)-6 levels in patients with COPD were elevated compared with HVs (1.5±1.06 pg/mL [mean ±1 SD] vs 0.4±0.24 pg/mL). LPS challenge increased the circulating IL-6 levels (7.5±2.72 pg/mL) 9 hours post challenge.

**Conclusions:**

This study shows the ability to quantify ‘whole lung’ neutrophil accumulation in HVs following LPS inhalation and in subjects with COPD using autologous radiolabelled neutrophils and SPECT/CT imaging. Moreover, the reproducibility observed supports the feasibility of using this approach to determine the efficacy of therapeutic agents aimed at altering neutrophil migration to the lungs.

**Key messages**

**What is the key question?**

- Can we quantify blood neutrophil clearance to the lung in (1) stable COPD and (2) lipopolysaccharide (LPS)-induced neutrophilic inflammation using sequential single-photon emission CT/CT?

**What is the bottom line?**

- Increased blood neutrophil clearance to the lung was observed both in patients with COPD and in LPS-challenged healthy volunteers (HVs) compared with saline-challenged HVs.
- This signal was reproducible over time and confirms the stability of the clearance value.

**Why read on?**

- This non-invasive platform has the potential to allow direct assessment of drugs that alter neutrophil kinetics and to support proof-of-concept studies.

## Introduction

COPD is a complex and heterogeneous condition in which neutrophils play a central role.^1^ Studying neutrophil accumulation across all lung compartments is challenging due to a lack of direct ‘whole lung’ assessment techniques. The aim of this study was to validate a new non-invasive method for measuring whole lung neutrophil accumulation in patients with stable COPD and in healthy volunteers (HVs) following both (1) inhaled lipopolysaccharide (LPS) and (2) inhaled saline.

Neutrophil granules contain an array of highly histotoxic molecules which have been shown in a number of models to induce airway inflammation, airway remodelling and glandular hypertrophy.^1^ In smokers, the extent of airway neutrophilia correlates with the severity of airflow limitation,^2^ and sputum neutrophil numbers predict disease progression.^3^ Moreover, patients lacking alpha-1-antitrypsin, the major serine protease responsible for neutralising neutrophil elastase, suffer severe and early-onset emphysema.^4^

Quantifying lung neutrophil load is currently restricted to counting neutrophil numbers in sputum and bronchoalveolar lavage fluid (BALF). However, these values can be highly variable and only reflect the airway and alveolar compartments.^5^ Hence, there is significant value in investigating approaches that could quantify whole lung neutrophil migration; this would increase our understanding of the pathophysiology of neutrophilia in lung disease and provide a novel approach for preclinical evaluation of therapies aimed at modulating neutrophil accumulation.

Neutrophilic inflammation can be induced using the bacterial cell wall component LPS, which when injected or inhaled triggers an intense inflammatory response.^6 7^ Systemic exposure to LPS causes transient fever and increased levels of circulating neutrophils and inflammatory mediators.^8–11^ Furthermore, inhaled LPS has been shown to increase neutrophil numbers in sputum by 22% 6 hours post challenge.^12^ Hence LPS administration to the lung provides a useful tool to induce neutrophil recruitment in an experimental setting.

In a preliminary study, we used single-photon emission CT (SPECT) to measure the uptake of autologous ^99m^Technetium (Tc)-labelled neutrophils in human lungs.^13^ A critical step has been the development of neutrophil isolation and labelling techniques that do not cause ex vivo activation of these cells, ensuring that they behave in a physiological manner when reinjected.^14 15^

The aim of the present study was to validate this non-invasive SPECT/CT technique by measuring the rate of neutrophil clearance from the blood compartment to the lungs (‘neutrophil clearance’) in HVs following LPS inhalation and by comparison in patients with clinically stable COPD. To assess the reproducibility of this index of lung neutrophil accumulation, a subset of patients with COPD were restudied after 7–10 days. Comprehensive circulating biomarkers were captured throughout to try to identify a systemic marker capable of predicting lung neutrophil accumulation.

## Methods

### Patient and healthy subject populations

Two groups, aged 45–75, were recruited. Group 1 consisted of non-smoking HVs and group 2 patients with stable COPD (Global initiative for chronic obstructive lung disease (GOLD) stages 2 and 3).^16^ Forty-three subjects were recruited and are included in the study population summaries, and 38 subjects were included in the per-protocol analysis. Details of the inclusion/exclusion criteria are provided in the online supplementary material.

10.1136/thoraxjnl-2018-212509.supp1 Supplementary file 1

### Study design

**Figure 1:**
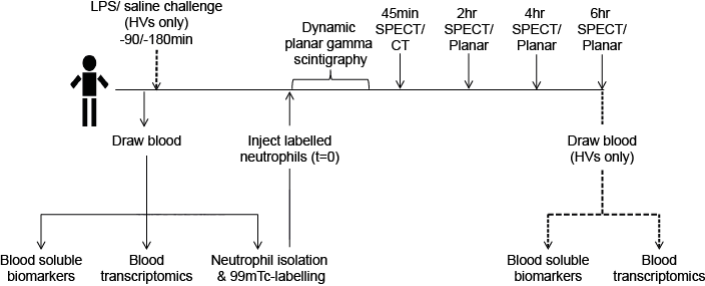
Schematic diagram of the study design. Eighteen healthy volunteers randomised 2:1 to receive LPS or saline and 20 patients with stable COPD (stage 2/3) completed the protocol. Ten patients with COPD were scanned 7–10 days later to determine reproducibility. ^99m^Tc, ^99m^Technetium; HVs, healthy volunteers; LPS, lipopolysaccharide; SPECT, single-photon emission CT.

Figure 1 shows a schematic of the study design. Visit 1 (V1) occurred within 4 weeks of screening. HVs were randomised (single-blind) at a ratio of 2:1 to receive inhaled LPS (50 µg) or saline via a breath-activated nebuliser either 90 min or 180 min before injection of ^99m^Tc-labelled neutrophils. Further study details are included in the online supplementary material.

### Neutrophil isolation and radiolabelling

Venous blood (80 mL) was anticoagulated with acid-citrate dextrose (1.5 mL/10 mL blood) and neutrophils (>95% purity) isolated using discontinuous Percoll plasma gradients.^17^ Radiolabelling was achieved using ^99m^Tc-hexamethylpropyleneamine oxime. Administered activities were 118–200 MBq (maximum dose of 2 mSv) with injected neutrophil numbers typically 70×10^6^.

### Gamma scintigraphy

Subjects were positioned supine in a double-headed gamma camera (GE Discovery 670). Activities in the thorax/upper abdomen were recorded using dynamic planar gamma scintigraphy (frame time 1 s for 2 min, then 20 s for 38 min). Additional 20 min static planar scans were obtained at 2 hours, 4 hours and 6 hours. SPECT images were obtained at 45 min (coupled with a low-dose [3 mSv] lung CT for regions of interest [ROI] definition), 2 hours, 4 hours and 6 hours (SPECT acquisition time 24 min). Rapid peripheral blood sampling was performed to assess radioactivity levels and differential white cell counts. A subgroup (n=10) of subjects with COPD returned 7–10 days later for a repeat study.

### Circulating biomarkers

Plasma, serum and whole blood samples were used for biomarker analysis (see online supplementary material).

### Labelled neutrophil recovery

The percentage of radiolabelled cells in the circulation 45 min postinjection (‘neutrophil recovery’) was assessed to ensure that in each study the cell isolation/labelling procedure had not caused cell activation. This value was calculated as: *(Radioactivity in sample [kBq] / [injected activity (kBq)/total blood volume (mL)]) × 100%*; blood volume was estimated using height and weight.^18^

### Dynamic scan analysis

ROIs were drawn over the right and left lungs, liver and spleen using Xeleris software (GE Healthcare). Mean counts/voxel were recorded, decay-corrected, and organ-time activity curves were generated.

### SPECT analysis

The Xeleris software was used to draw ROIs over reconstructed right and left SPECT slices at each time-point (online supplementary figure E1) avoiding the mediastinum and areas of lung close to the liver and spleen where there was scatter from physiological uptake of neutrophils. Small areas within the posterior lower lobes corresponding to CT-confirmed areas of postural-dependent change were also excluded. Data were decay-corrected for ^99m^Tc and mean count/voxel for each lung volume was determined. Patlak-Rutland graphical analysis (see online data supplementary material for further details) was applied to determine time-dependent blood neutrophil clearance (mL/min/mL lung blood distribution volume).^19^ Venous blood activities formed the input function.

10.1136/thoraxjnl-2018-212509.supp2 Supplementary file 2

### Statistical analysis

Results are presented as mean ±1 SD; statistical analysis is as detailed in the figure legends.

## Results

### Patient demographics and safety data

Subject demographics are provided in table 1. The predominance of men in the group of patients with COPD reflects the demographics of this condition.^20^ HVs were non-smokers and slightly younger than the subjects with COPD. The COPD cohort included current and ex-smokers. Adverse events were minor (online supplementary table E1). One subject with COPD was withdrawn due to vomiting deemed to be unrelated to the study. Imaging identified an asymptomatic lung cancer in one patient with COPD, which was subsequently resected.

**Table 1:** Demographic information

Demographics	All COPD (n=21), mean (SD)	All HV (n=22), mean (SD)
Age (years)	67.4 (3.4)	61.0 (8.6)
Sex, n (%)		
Female	5 (24)	9 (41)
Male	16 (76)	13 (59)
BMI (kg/m^2^)	25.5 (3.8)	25.9 (2.8)
Height (cm)	169.9 (6.9)	171.4 (8.9)
Weight (kg)	73.9 (12.8)	76.1 (10.1)
FEV_1_ (L)	1.7 (0.4)	3.2 (0.7)
FEV_1_ (%)	61.9 (11.3)	–
FEV1:FVC ratio	52.3 (9.4)	–
ICS usage, n (%)	18 (86)	0 (0)
Current smoker, n (%)		
Yes	4 (19)	0 (0)
No	17 (81)	22 (100)
Years smoked	37.1 (10.8)	–
Pack years	43.9 (20.3)	–

Forty-three subjects were recruited into the study and are included in the population summary; however, only 38 subjects were included in the per-protocol efficacy/data analysis. No significant difference in the demographics of patients analysed per-protocol was noted.

Three HVs did not undergo SPECT/CT due to failures with either (1) the neutrophil labelling process or (2) nuclear medicine equipment. The dose of inhaled LPS was below the set 50 µg in one HV and therefore data were excluded from per-protocol analysis. One subject with COPD became ill and was withdrawn prior to initiation of imaging.

BMI, body mass index; HV, healthy volunteer; ICS, inhaled corticosteroid; LPS, lipopolysaccharide; SPECT, single-photon emission CT.

### ^99m^Tc-labelled neutrophil recovery

Forty-five-minute blood recovery values for the injected radiolabelled neutrophils in both COPD and HVs were in line with previous results (online supplementary figure E2A).^13 21^ This confirmed that our isolation/labelling procedures did not activate neutrophils. Of note, neutrophil recoveries in the COPD group (V1: 39.1%±12.5; visit 2 (V2): 39.1%±8.9) were higher than in either the LPS-challenged (31.7%±7.2;) or saline-challenged (30.8%±4.1) HVs. This agrees with our SPECT/CT findings (online supplementary figure E2B) where we noted a higher intensity in the SPECT mediastinal vascular signal at 45 min in patients with COPD.

10.1136/thoraxjnl-2018-212509.supp3 Supplementary file 3

### Systemic effect of inhaled LPS

The biological impact of LPS was verified by the marked rise in blood neutrophil count (online supplementary figure E2C). Data are consistent with previous observations^22^ and show a significant 2.1-fold increase 330 min post 50 µg inhaled LPS, increasing to 2.3-fold at 450 min. Neutrophil counts were consistent throughout in saline-challenged HVs. A modest rise in body temperature was seen in HVs who inhaled LPS compared with saline (LPS 0.68°C±0.33°C, saline 0.18°C±0.44°C at 360 min, p=0.02). The extent of blood neutrophilia observed aligns with that seen following intravenous LPS administration.^23^

### Dynamic and static gamma scintigraphy

The data generated from the planar images were expressed as decay-corrected organ counts/pixel/MBq against time (online supplementary figure E3). The area under the curve (AUC) values are proportional to the size of the marginating neutrophil pools in each organ and reflects changes in neutrophil biodistribution. Similar results were observed between COPD, LPS HVs and saline HVs in the immediate lung time–activity curves (online supplementary figure E3A–B). However, the early splenic marginated neutrophil pool, as calculated by AUC in first 40 min, was greater in patients with COPD (V1) (20.8±7.0 counts/pixel/MBq.min) than in LPS-challenged (15.8±4.7 counts/pixel/MBq.min) or saline-challenged (12.3±5.4 counts/pixel/MBq.min) HVs and remained so 6 hours post injection (online supplementary figure E3C). ^99m^Tc-neutrophil accumulation was higher in the liver in COPD and LPS-treated HVs compared with saline-treated HVs (online supplementary figure E3D), which may reflect a reduction in bone marrow pooling.

10.1136/thoraxjnl-2018-212509.supp4 Supplementary file 4

### Blood ^99m^Tc-neutrophil clearance to the lungs

**Figure 2:**
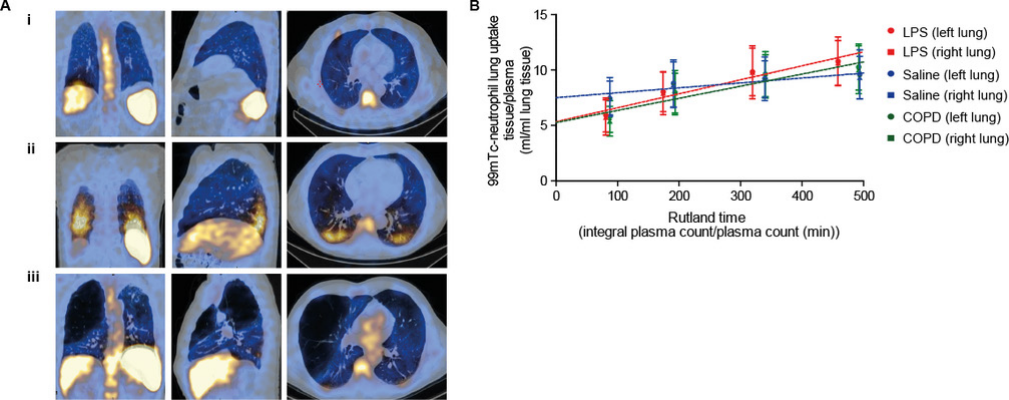
SPECT/CT and Patlak-Rutland analysis. Panel A shows a two-dimensional representation (coronal, sagittal and transverse views, respectively) of a reconstructed 45 min SPECT/CT scan in (i) a saline-challenged HV, (ii) an LPS-challenged HV and (iii) a patient with COPD. Peak areas of radioactivity are seen as yellow/white, with lower levels in blue. The large airspaces, with negligible radioactivity, are black and can be seen in the emphysematous lung (iii). Panel B shows the composite Patlak-Rutland graphical plot (±SD) in saline- challenged HVs (n=6), LPS-challenged HVs (n=12) and patients with COPD (n=20) (V1). For visual purposes the right and left lungs have been displayed separately. The plot gradient represents blood clearance of ^99m^Tc-neutrophils to the lungs in mL/min/mL lung volume. The y-axis intercept corresponds to the ^99m^Tc-neutrophil distribution volume. 99^m^Tc, ^99m^Technetium; HV, healthy volunteer; LPS, lipopolysaccharide; SPECT, single-photon emission CT.

Figure 2A shows representative 45 min SPECT images overlaid with coronal, sagittal and transverse CT images in (1) saline-challenged HVs, (2) LPS-challenged HVs and (3) patients with COPD, and illustrates the normal physiological accumulation of ^99m^Tc-neutrophils to marginated pools within the liver, spleen and to a lesser extent the bone marrow (as shown by the vertebral bone marrow signal).

There were a number of notable differences in the appearance of the SPECT images in subjects inhaling LPS and patients with COPD compared with saline-treated HVs. The most evident was the intense bilateral posterior-basal signal, which corresponded to areas of posture-dependent parenchymal change seen on CT most likely resulting from the enhanced hold-up of neutrophils in the pulmonary microvasculature in areas of lung atelectasis induced by prolonged lying in the supine position (figure 2A). For this reason, these small areas were excluded in the ROI drawn on the reconstructed lungs. We also observed a reduction in the bone marrow signal in the LPS-treated HV group (figure 2Aii). The lung signal in patients with COPD was more heterogeneously distributed and absent in areas corresponding to large bullae (figure 2Aiii).

**Figure 3:**
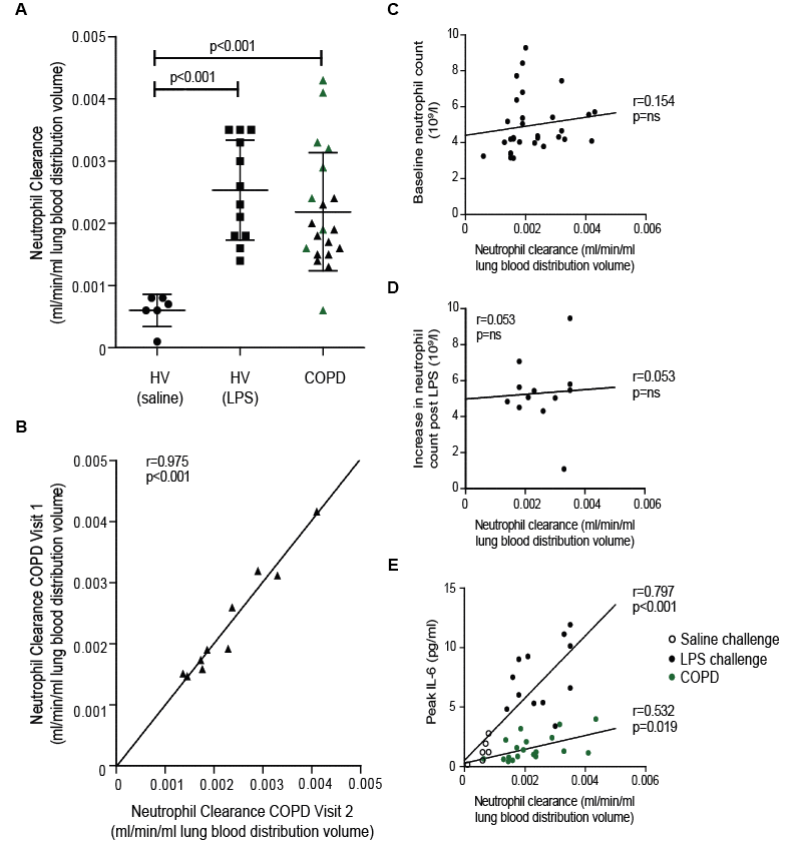
Neutrophil clearance data. Panel A shows ^99m^Tc-neutrophil clearance (slope/intercept) in each individual patient, with mean and SD for all saline-challenged HVs (n=6), LPS-challenged volunteers (n=12) and patients with COPD (V1) (n=20). Green triangles represent patients with a diagnosis of chronic bronchitis. Non-parametric tests were used to compare groups (only significant p values are presented). Panel B displays the correlation in neutrophil clearance between the two studies in patients with COPD with repeat scans. No correlation was observed between neutrophil clearance and baseline neutrophil counts in COPD (panel C) or the absolute increase in circulating neutrophil counts post-LPS (panel D). The absolute neutrophil count prior to LPS challenge was 3.9±1.2 10×9/L. Panel E shows clearance values in relation to (1) peak circulating IL-6 levels in saline HVs (open circles) and LPS HVs (closed circles) and (2) baseline IL-6 levels in patients with COPD (green circles). HVs and patients with COPD were analysed independently with results for each group displayed. Correlations for all data sets were determined using a Pearson correlation coefficient and are displayed on each data set along with the corresponding p value. ^99m^Tc, ^99m^Technetium; HV, healthy volunteer; IL-6, interleukin 6; LPS, lipopolysaccharide.

Patlak-Rutland graphical analysis was used to quantify neutrophil accumulation in the lungs, based on the four sequential SPECT images performed in each subject (figure 2B). This represents the clearance of ^99m^Tc-neutrophils from the circulating blood pool to the lungs, with neutrophil uptake plotted against ‘normalised’ time (integral of plasma counts/instantaneous plasma counts [min]). The gradient of the slope corresponds to the neutrophil clearance from the blood into the lungs and the intercept the volume of distribution of the labelled cells. The slope/intercept therefore expresses neutrophil clearance in terms of distribution volume. Figure 3A shows individual subject data, and only single visit data (V1) were included in the COPD cohort. Neutrophil clearance was significantly greater in patients with COPD (V1) (0.0022±0.0010 mL/min/mL) and LPS-challenged HVs (0.0025±0.0008 mL/min/mL) compared with saline-challenged controls (0.0006±0.0003 mL/min/mL) (p=0.003 and p<0.001, respectively). Moreover, in the COPD group, a diagnosis of chronic bronchitis (Medical Research council (MRC) criteria, shown as green symbols in figure 3A) was associated with a higher lung neutrophil clearance compared with non-sputum producers (0.0027±0.0012 vs 0.0018±0.0004 mL/min/mL) (p<0.05).

A technical caveat with the above analysis was that the ROIs were drawn separately at each study time-point, which led to some differences in the number of lung slices being included at different time-points. This was driven principally by the time-dependent fall in the intensity of the liver and spleen signals allowing a greater volume of lung to be included in the later scans due to lack of apparent spillover. To exclude this as a potential source of bias, an alternative approach involving coregistration of the images from all time-points and placement of a smaller but fixed ROI in the lung, thus allowing analysis of identical regions in all SPECTs, was implemented (online supplementary figure E4). This approach fully supported our original findings.

10.1136/thoraxjnl-2018-212509.supp5 Supplementary file 5

### Reproducibility of ^99m^Tc-neutrophil clearance to the lungs

To explore the reproducibility of our analysis, a cohort of 10 subjects with COPD were restudied 7–10 days later. Figure 3B shows the neutrophil clearance values obtained for these subjects at both visits. Despite the intersubject variability, there was striking intrasubject reproducibility of the signal, with a correlation of r=0.975 (p<0.001) and no difference in the mean neutrophil clearance values between the two imaging visits (vV1: 0.0022±0.0010 mL/min/mL, V2: 0.0023±0.0009 mL/min/mL, p=0.999).

### Correlation of ^99m^Tc-neutrophil clearance with circulating biomarkers

Analysis of whole blood mRNA transcriptomic changes (online supplementary figure E5A) revealed a large number of differentially expressed genes in patients with COPD versus HVs (99 genes altered with fold change >1.5, unadjusted p<0.05). These were consistent with mRNA profiles reported elsewhere.^24 25^ Significant changes were also seen in blood samples in HVs after saline and LPS challenge (saline evoked 113 and LPS evoked 185 genes with fold change >1.5, unadjusted p<0.05); the genes altered by LPS were associated with infection biology pathways (Toll-like receptors and mammalian target of rapamycin (mTOR) pathways), whereas those changed by saline did not map to any coherent biological pathways (data not shown). There was no overlap between the blood mRNA transcript changes evoked by LPS and those seen in the subjects with COPD. These data highlight that while LPS inhalation can induce a similar degree of neutrophil accumulation in the lungs to that observed in COPD, the effect may be driven by a distinct pathophysiological mechanism.

10.1136/thoraxjnl-2018-212509.supp6 Supplementary file 6

To explore whether a circulating biomarker might predict lung neutrophil clearance, blood samples were analysed for a variety of soluble mediators. Baseline neutrophil counts were higher in subjects with COPD compared with HVs (p=0.004), but there was no difference in blood eosinophil counts (p=0.10; online supplementary figure E5C). There was no correlation between absolute blood neutrophil or eosinophil counts (or incremental increases) and ^99m^Tc-neutrophil clearance in the lungs in any of the groups (p>0.05 in all cases). Moreover, no correlation was observed between neutrophil clearance and total increase in neutrophil count from baseline to peak in the LPS-challenged group (figure 3C). Circulating interleukin (IL)-6 levels correlated with neutrophil clearance in both the HV and COPD cohorts (figure 3D).

The levels of circulating IL-6, fibrinogen and tumour necrosis factor (TNF) were higher in the subjects with COPD (V1) compared with baseline values for both HV groups (online supplementary figure E5B; COPD vs LPS-treated HVs: p<0.001, p=0.005 and p<0.001, respectively; COPD vs saline-treated HVs: p=0.003, p=0.024 and p=0.017, respectively). The levels of monocyte chemoattractant protein (MCP2) were also higher in subjects with COPD compared with subjects in the LPS-treated HV group (p=0.042). Baseline C-reactive protein (CRP), stomal cell-derived factor 1 (SDF-1) and IL-8 levels were similar between the groups. Of note, however, LPS challenge led to a significant increase in circulating IL-6 levels (online supplementary figure E6A; p=0.001), and a correlation was seen between ^99m^Tc-neutrophil clearance in the lungs and baseline circulating levels of IL-6 in COPD (p=0.019) and peak IL-6 in the HVs (p<0.001) (figure 3E). None of the other circulating biomarkers correlated with neutrophil clearance (online supplementary figure E7).

10.1136/thoraxjnl-2018-212509.supp7 Supplementary file 7

10.1136/thoraxjnl-2018-212509.supp8 Supplementary file 8

## Discussion

There is a need for new non-invasive techniques to support early-phase clinical studies in COPD, in particular methodologies that test the efficacy of drugs targeting neutrophilic inflammation. Existing approaches are crude and focus on neutrophil counts in sputum, BALF and airway biopsies,^26 27^ all of which have significant limitations. This study has used the Patlak-Rutland graphical analysis of sequential ^99m^Tc-labelled neutrophil images to calculate the clearance of neutrophils into the lungs of human subjects. We observed a highly reproducible increase in neutrophil clearance in patients with COPD and a similar increase in neutrophil clearance in healthy subjects following inhalation of LPS. The assessment procedures used in this study were non-invasive and well tolerated. This result confirms the findings of our earlier pilot study^13^ and suggests that patients with chronic bronchitis may have an even higher level of blood neutrophil clearance in their lungs compared with non-productive COPD patients.

The data obtained from the cohort of patients with COPD restudied after 7–10 days showed the highly reproducible nature of this SPECT/CT imaging technique. This level of reproducibility and signal stability is far better than that typically offered by sputum-based measurements, which show significant day–day variability, and may be of particular value when designing intervention studies with novel therapeutics. A power calculation showed that eight subjects would result in 90% power to detect a true reduction of 50% in lung neutrophil clearance at the 5% level of significance using a two-sided paired t-test. This assumes an SD of differences on the log scale of 0.5 and a mean of paired differences of −0.7 on the log scale (equivalent to a 50% reduction).

The high percentage of inhaled corticosteroid (ICS) usage in our COPD population is worth noting and may have affected the enhanced neutrophil clearance we observed; however, the exact relationship between ICS use and neutrophil clearance would need to be the subject of future studies. In addition, our inhaled LPS model would allow for the effects of varying doses of ICS to be analysed. There are also potential benefits in also using this platform to determine the dynamics of neutrophil clearance during both infective and non-infective exacerbations of COPD. Given our group has previously demonstrated an accumulation of ^99m^Tc-neutrophils to an area of pneumonia,^28^ we would anticipate an overall increase in lung neutrophil clearance with potential ‘hotspots’ over evolving areas of infection.

LPS inhalation is a well-established method to induce acute neutrophilic inflammation in the lung, and previous studies have demonstrated rapid increases in sputum and BALF neutrophil counts following such challenges.^12 29^ Our study shows for the first time the magnitude and regional distribution of this effect, with LPS causing a clear and consistent increase in the accumulation of ^99m^Tc-labelled neutrophils in the lung. The early entrapment of radiolabelled neutrophils observed in the posterior lung bases most likely reflects early vascular hold-up of LPS-primed neutrophils^30^ in relatively compressed regions of the lung and hence not thought to be representative of neutrophil accumulation in normally ventilated lung.

The Patlak-Rutland graphical analysis would give neutrophil clearance in mL/min if the absolute concentration of ^99m^Tc in the lungs could be measured, but this is currently not possible with SPECT. Normalising clearance to distribution volume is a valid way of overcoming this deficiency provided the distribution volume does not vary between patient groups. In the current context, the distribution volume effectively represents the population of labelled neutrophils present in the lungs before any have migrated into the extravascular space, and therefore the total pulmonary blood circulating pool. The distribution volume would therefore be expected to be expanded if labelled neutrophil transit through lung capillaries was delayed, such as occurs when they are activated or primed. It is reasonable to assume, however, that such activation would not be greater in unchallenged HVs, thereby making it correspondingly unlikely that low neutrophil clearance in such HVs compared with the other two groups is an artifice resulting from a greater volume of distribution. In any event, there was no significant difference in lung intravascular transit of labelled neutrophils between the three patient groups.

There are a number of papers indicating an alteration in ex vivo neutrophil function in patients with COPD, including migration, phagocytosis, reactive oxygen species generation, degranulation and extracellular trap production.^31 32^ Nevertheless, there is very little information on neutrophil kinetics or the in vivo biodistribution of neutrophils in this condition. Historically this has been challenging due to the difficulties of maintaining neutrophils in a non-activated state during isolation and radiolabelling. Further studies to determine the mechanism of the observed increase in marginating neutrophils in both the liver and spleen in COPD are warranted. The physiological distribution of intravascular neutrophils in healthy individuals partitions equally between marginating and circulating pools, and LPS has been shown to increase the size of both pools.^33^ Moreover recent studies would support a link between the rise in IL-6 and the blood neutrophilia observed post-LPS.^34 35^ Our data also show that inhaled LPS induces an increase in the accumulation of neutrophils in the spleen, and to a lesser extent the liver; however, whether this reflects enhanced uptake by these organs, or an indirect effect of LPS suppressing neutrophil margination in bone marrow, requires further investigation.

As anticipated, we observed increased levels of IL-6, fibrinogen and TNF in the circulation of our subjects with COPD compared with HVs together with a higher circulating neutrophil count. These data support the concept that COPD is a systemic inflammatory disorder.^36^ Levels of circulating IL-6 also increased following LPS challenge and again correlated with lung neutrophil clearance. The lack of correlation of neutrophil clearance to some of the other markers we measured post-LPS may reflect our limited sampling times, sample size and/or the relatively low dose of LPS used.^37^ Circulating IL-8 and TNF levels in particular have been shown to peak around 4 hours post-LPS, with return to baseline by 6 hours.^23^

Of note, our biomarker analysis revealed changes in gene expression in HVs following both inhaled saline and LPS. While these changes were expected in the LPS group, such alterations in the saline group were unanticipated. This is an interesting observation and requires replication of this data set in a second independent study.

We acknowledge that an in situ labelling technique^38^ would be much simpler than the autologous radiolabelling and reinfusion of cells used in this study. However, it is important to understand that if such a technique were to emerge, any suitable tracer would need to be stable, non-toxic and highly selective for neutrophils, and only label cells present in the freely circulating vascular pool. Moreover, the predicted lower concentrations of label that might be used for any in situ labelling approach might reduce the sensitivity compared with the current method of ex vivo labelling. Hence the two techniques provide different information and can be considered to be complementary.

In summary this study reports an entirely new approach to quantifying neutrophil accumulation in the lungs of patients with COPD by coupling autologous ^99m^Tc-labelled neutrophils to SPECT/CT imaging. Widespread uptake of this imaging technique by other centres would require training in neutrophil isolation techniques and cell labelling facilities, and access to appropriate SPECT/CT facilities. The extent of neutrophil accumulation in the lung appears to correlate with circulating IL-6 levels and be more marked in those with a chronic bronchitis phenotype. Our studies also provide new insights into neutrophil kinetics generally in patients with COPD, with altered neutrophil distribution between the bone marrow, liver and splenic pools. This imaging platform provides a new quantitative and non-invasive way to assess neutrophil accumulation in the lungs of healthy or disease subjects. Moreover, the reproducibility of this technique should support its use to examine the efficacy of therapeutic agents aimed at reducing neutrophilic inflammation.
