# Supplementary material for: Use of autologous 99mTechnetium-labelled neutrophils to quantify lung neutrophil clearance in COPD
Source: Thorax. 2019 Jan 23;74(7):659–66. doi: 10.1136/thoraxjnl-2018-212509 (PMC6585304; doi:10.1136/thoraxjnl-2018-212509)
Supplement: Supplementary file 1 [file thoraxjnl-2018-212509supp001.pdf]

Use of autologous ^{99m}Technetium-labelled neutrophils to quantify lung neutrophil clearance in COPD

Tregay N[1], Begg M[2], Cahn A[3], Farahi N[1], Povey K[4], Madhavan S[5], Simmonds R[1], Gillett D[6], Solanki C[6], Wong A[6], Maison J[5], Lennon M[7], Bradley G[7], Jarvis E[8], de Groot M[9,10], Wilson F[9], Babar J[10], Peters AM[11], Hessel EM[2], Chilvers ER[1]

[1]Department of Medicine, University of Cambridge, UK
[2]Refractory Respiratory Inflammation DPU, Respiratory TAU, GlaxoSmithKline, Stevenage, UK
[3]Discovery Medicine, Respiratory TAU, GlaxoSmithKline, Stevenage, UK
[4]Clinical Pharmacology Science & Study Operations, GlaxoSmithKline, Stockley Park, UK
[5]Clinical Unit Cambridge, GlaxoSmithKline, Cambridge, UK
[6]Department of Nuclear Medicine, Cambridge University Hospitals, Cambridge UK
[7]Target Sciences, GlaxoSmithKline, Stevenage, UK
[8]Biostatistics, GlaxoSmithKline, Stevenage, UK
[9]Experimental Medicine Unit, Immuno-Inflammation TAU, GlaxoSmithKline, Stevenage, UK
[10]Department of Radiology, Cambridge University Hospitals, Cambridge UK
[11]Division of Clinical and Laboratory Investigation, Brighton and Sussex Medical School UK

Online Data Supplement

METHODS

Patient Population

Group 1 consisted of HVs with <5 pack year smoking history and no cigarettes smoked within 6 months of study commencement, with a body weight ≥50kg and body mass index (BMI) of 19-30kg/m^2. HVs were excluded if there was a history of any acute lower respiratory tract illness within 4 weeks of screening, a current diagnosis of asthma, excluding childhood asthma, or an abnormal spirometry result defined as a forced expiratory volume in 1 second (FEV_1) ≤80% of predicted or a FEV_1 to forced vital capacity (FVC) ratio ≤70%.

Group 2 consisted of stable COPD patients (GOLD stage 2 or 3)(1) with a body weight ≥45kg and a BMI 18-32kg/m^2. Patients with stage 3 disease but an FEV_1 <40% predicted were excluded to avoid breathing difficulties associated with prolonged periods of lying. Patients with a second respiratory diagnosis, a systemic inflammatory condition, any co-morbidity where neutrophil function is known to be affected, or untreated anaemia were also excluded. Use of oral steroids within 3 months of the study or monoclonal antibody therapy within 12 months of the study was prohibited. COPD was considered clinical stable if there had been no change in clinical symptoms in the three months prior to screening. Post-hoc analysis was performed to investigate differences between COPD patients with, and without, chronic bronchitis.

Study Design

Data from an initial HV cohort (n=3 per group) were used to explore neutrophil clearance in the lung following LPS administered either 90 or 180min prior to the injection of radiolabelled neutrophils and the initiation of imaging. These time-point options were proposed based on the logistical constraints of the cell isolation and labelling process and the predicted time-course reported for LPS-induced neutrophil accumulation in the lung in studies using bronchoscopy and BAL to assess neutrophil influx(2). Given that the enhanced signal observed was identical for LPS administered at either -90 or -180min the remainder of the LPS-challenge cohort (n=6) received the LPS challenge 90min prior to injection of the radiolabelled neutrophils. Data were combined for analysis purposes. For all subjects vital statistics and adverse events (AEs) were recorded throughout the study, either volunteered by the subjects or through investigator questioning.

Circulating biomarkers

Blood samples for biomarker analysis were taken at baseline in our group 2 (COPD) subjects and in group 1 (HVs) 180min and 60min before and 360min post injection of the ^{99m}Tc-neutrophils. Biomarker data was obtained from all subjects enrolled in the study regardless of whether they completed the scanning protocol (Saline: n=7; LPS: n=14; COPD: n=-21). Plasma was prepared from 2ml whole blood anticoagulated with sodium citrate, which was centrifuged at 4°C, 1500*g* for 15min. Fibrinogen was measured by ELISA. Serum was prepared from 8.5 ml whole blood in serum-separating tubes; samples were left to clot for 30min and centrifuged at RT for 10min at 1300*g*. C-Reactive protein (high-sensitivity assay), Interleukin 6 (IL-6), Interleukin 8 (IL-8), Interleukin 1β (IL-1β), Tumour necrosis factor alpha (TNF) (V-Plex Plus Human Proinflammatory Panel I 10 spot plate, MSD), Monocyte chemotactic protein -2 (MCP-2) (Human MCP-2 kit 4 spot plate, MSD), Stromal cell-derived factor 1 (SDF1) (Human CXCL12/SDF1α Immunoassay - Quantikine ELISA, R&D Systems: samples run 1 in 10 with a 1 in 10 dilution for plasma) and β-defensin (Human β-defensin 2 kit, My Biosource) were analysed from the prepared serum. Data were also generated for CCL16 (Human

CCL16 ELISA kit, My Biosource) but not reported due to a technical QC fail of the ELISA. Data for β-defensin and IL-1β are not presented as all values were below the lower limit of quantification for their respective ELISAs, data from all other assays were above the lower limit of detection. Whole blood was collected in 2.5ml PAXgene™ Blood RNA Tubes (BD Diagnostics), handled according to manufactures instructions. All samples were processed as a single batch at the end of the study. Serum and plasma biomarkers were analysed using parametric tests unless specified otherwise. RNA transcriptomics detection was conducted using HG-U133 Plus 2.0 GeneChip oligonucleotide arrays (Affymetrix, Santa Clara, CA, USA), and the data normalised, quality checked, and analysed as described)(3). One healthy volunteer sample (HVT_1330_-180 MIN_E4) failed QC and was excluded from the analysis. Transcript changes were considered significant if their fold change >1.5 and *P*-value <0.05. The data has been deposited in the National Center for Biotechnology Information Gene Expression Omnibus (GEO) and are accessible through GEO series accession number GSE112811. Baseline biomarker data was analysed post-hoc.

Patlak-Rutland analysis

Data from Patlak-Rutland graphical analysis are expressed as a Patlak plot (Figure 2 Panel B). The plot gradient (slope) represents neutrophil uptake into the lungs (per ml/min/ml lung volume) with the y axis intercept representing the initial lung blood distribution volume. Time-dependant neutrophil accumulation (clearance) is expressed as ml/min/ml of lung blood distribution volume, calculated by dividing the slope by the intercept. Results from each group are expressed in terms of mean ± 1 SD. Data between test and reference groups were compared using parametric tests for normally distributed data and non-parametric tests for non-normally distributed data, a *P*-value <0.05 taken as statistically significant.

References

E1 Tanday, S. Rethinking COPD diagnosis: imaging and GOLD criteria. *The Lancet Respiratory Medicine* 2015;15:doi:org/10.1016/S2213-2600.

E2 Wesselius, L. J., Nelson, M. E., Bailey, K., & O'Brien-Ladner, A. R. Rapid lung cytokine accumulation and neutrophil recruitment after lipopolysaccharide inhalation by cigarette smokers and nonsmokers. *Journal of Laboratory and Clinical Medicine* 1997;*129*:1:106–114.

E3 Juss, J., Herre, J., Begg, M., Bradley, G., Lennon, M., Amour, A., Chilvers, E. R. Genome-wide transcription profiling in neutrophils in acute respiratory distress syndrome. *Lancet* 2015*;*385:S55:doi:org/10.1016/S0140-6736(15)60370

TABLE E1

Adverse events: All adverse events related to the study are included

Preferred Term	All COPD N=21	All HV N=22	HV-LPS N=15	HV-Saline N=7
**Any AE [n (%)]**	**15 (71)**	**13 (59)**	**9 (60)**	**4 (57)**
Contusion	3 (14)	4 (18)	3 (20)	1 (14)
Musculoskeletal pain	5 (24)	0	0	0
Headache	2 (10)	2 (9)	2 (13)	0
Back pain	2 (10)	1 (5)	1 (7)	0
Fatigue	1 (5)	1 (5)	1 (7)	0
Nasopharyngitis	1 (5)	1 (5)	1 (7)	0
Epistaxis	0	1 (5)	0	1 (14)
Sneezing	0	1 (5)	0	1 (14)
Nausea	0	1 (5)	0	1 (14)
Decreased Appetite	0	1 (5)	0	1 (14)
Myalgia	0	1 (5)	1 (7)	0
Pain	0	1 (5)	1 (7)	0
Respiratory tract infection	0	1 (5)	1 (7)	0
Seasonal Allergy	0	1 (5)	1 (7)	0
Chest discomfort	1 (5)	0	0	0
Dizziness	1 (5)	0	0	0
Musculoskeletal discomfort	1 (5)	0	0	0
Gout	1 (5)	0	0	0
Presyncope	1 (5)	0	0	0

Pulmonary Mass	1 (5)	0	0	0
Rhinorrhoea	1 (5)	0	0	0
Vomiting	1 (5)*	0	0	0

*This subject was withdrawn due to this AE
