# Supplementary figures and images for: Use of autologous 99mTechnetium-labelled neutrophils to quantify lung neutrophil clearance in COPD
Source: Thorax. 2019 Jan 23;74(7):659–66. doi: 10.1136/thoraxjnl-2018-212509 (PMC6585304; doi:10.1136/thoraxjnl-2018-212509)

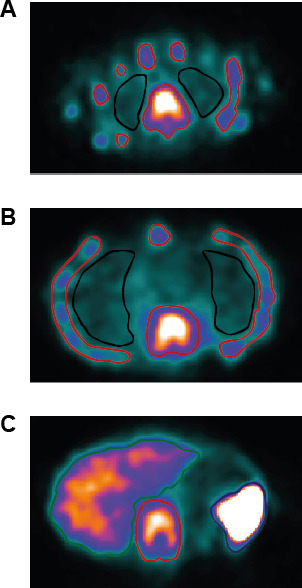

Supplement: Supplementary file 2 [file thoraxjnl-2018-212509supp002.jpg]

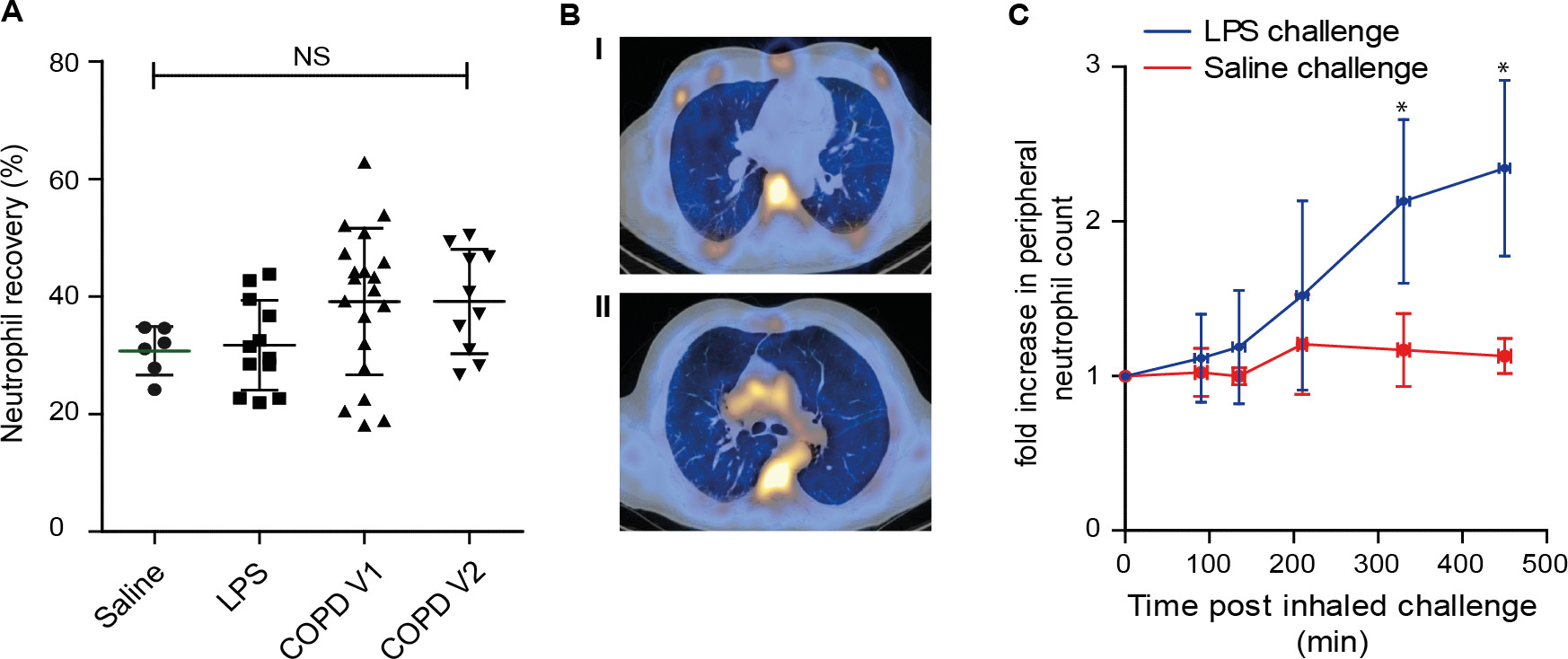

Supplement: Supplementary file 3 [file thoraxjnl-2018-212509supp003.jpg]

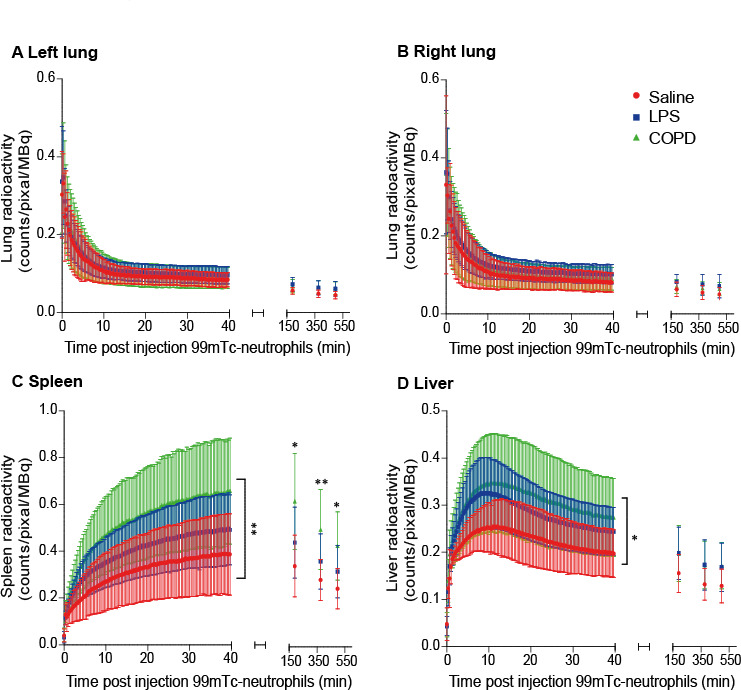

Supplement: Supplementary file 4 [file thoraxjnl-2018-212509supp004.jpg]

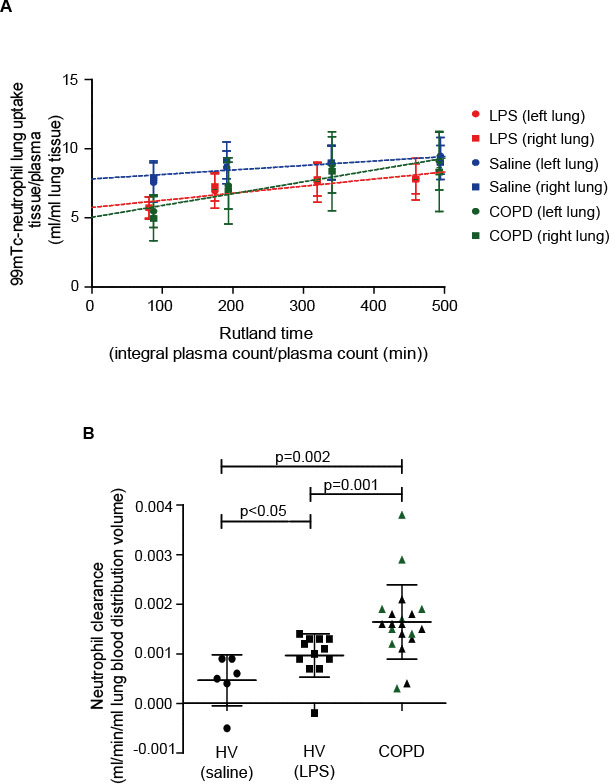

Supplement: Supplementary file 5 [file thoraxjnl-2018-212509supp005.jpg]

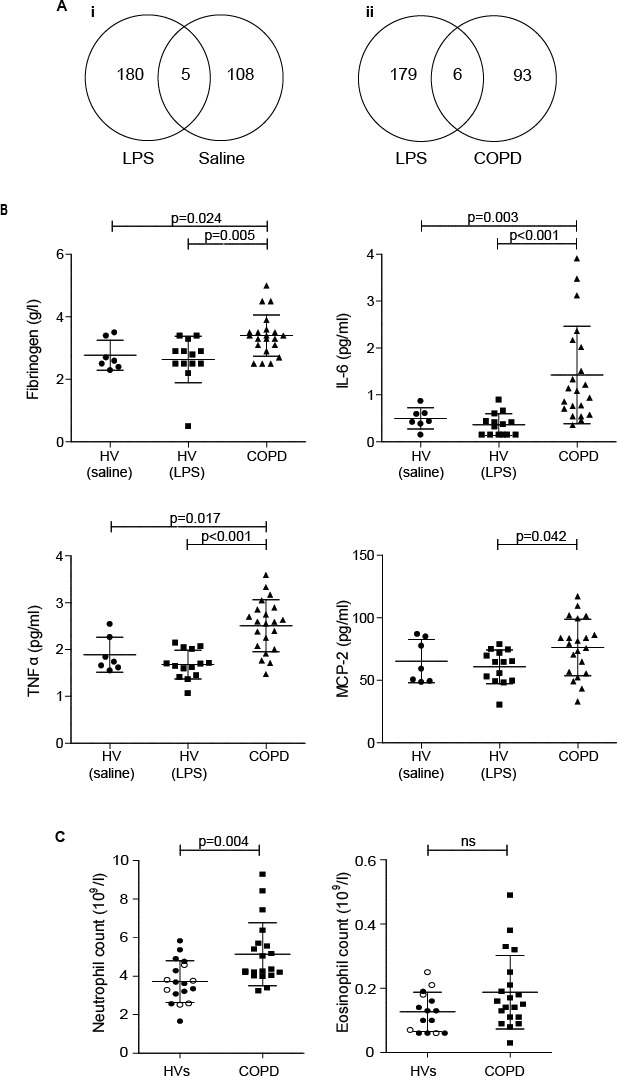

Supplement: Supplementary file 6 [file thoraxjnl-2018-212509supp006.jpg]

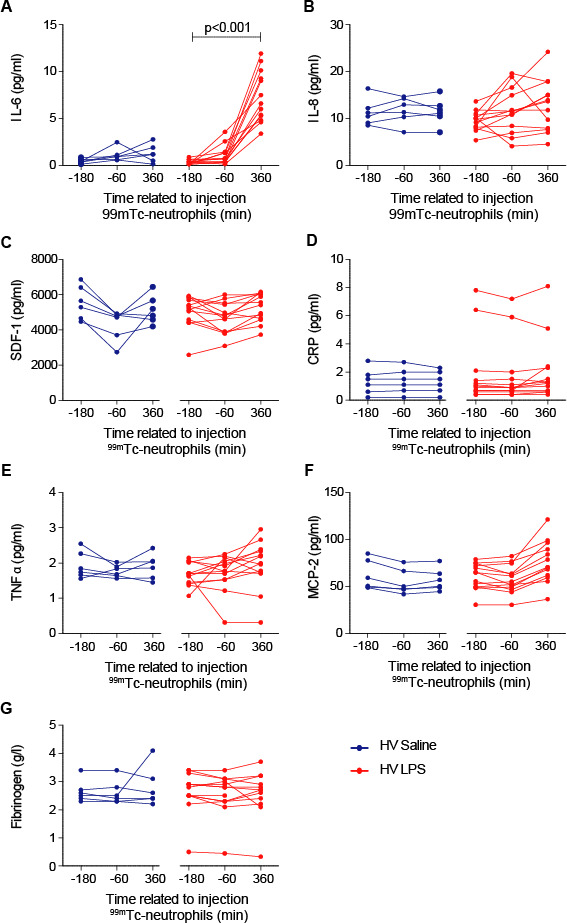

Supplement: Supplementary file 7 [file thoraxjnl-2018-212509supp007.jpg]

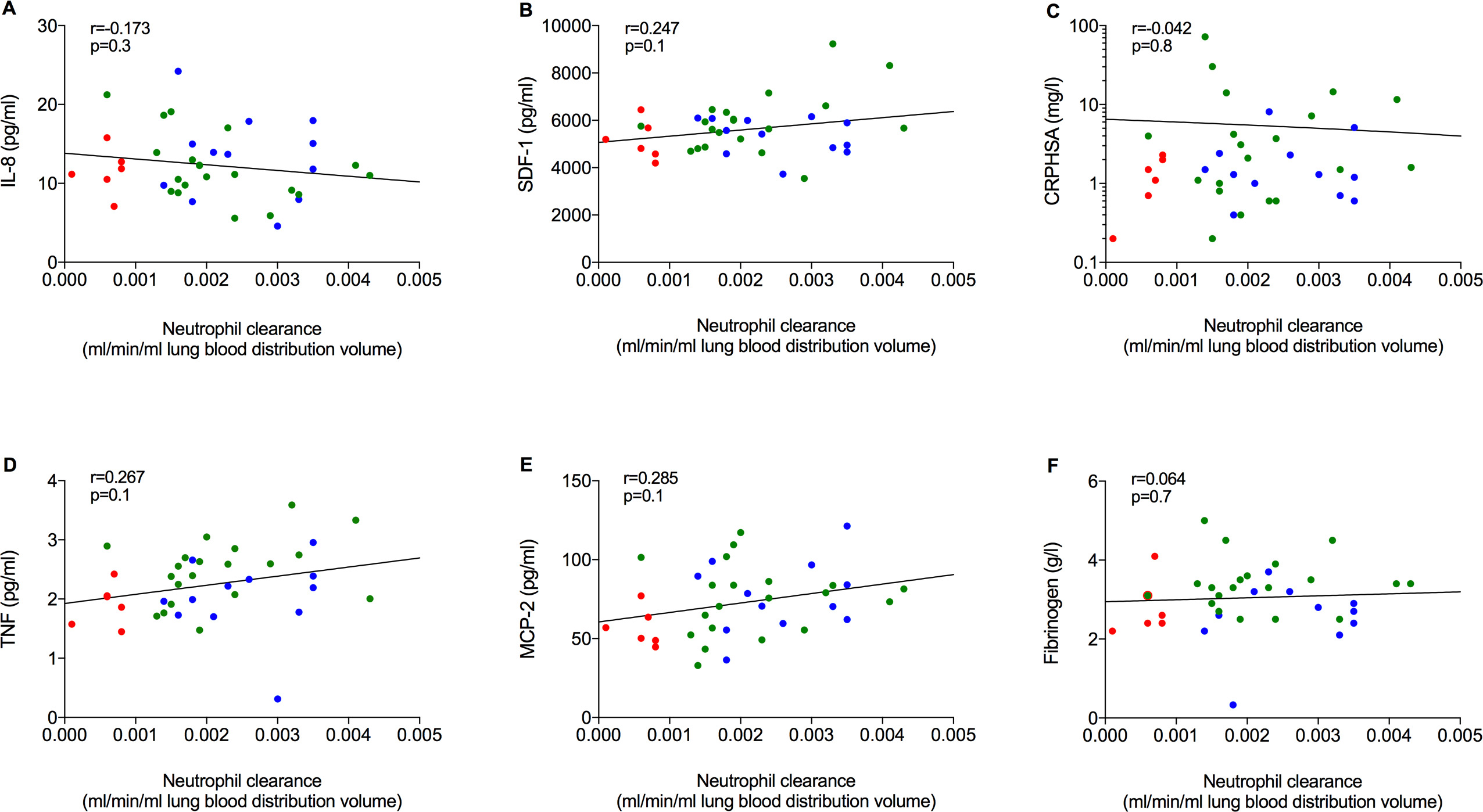

Supplement: Supplementary file 8 [file thoraxjnl-2018-212509supp008.jpg]
